# Intra- and Inter-Individual Differences in Adolescent Depressive Mood: the Role of Relationships with Parents and Friends

**DOI:** 10.1007/s10802-017-0321-6

**Published:** 2017-06-14

**Authors:** Shiyu Zhang, Laura Baams, Daphne van de Bongardt, Judith Semon Dubas

**Affiliations:** 1 Department of Developmental Psychology, Utrecht University, Utrecht, The Netherlands; 2 Population Research Center, University of Texas at Austin, Austin, TX USA; 3 Department of Psychology, Education and Child Studies, Erasmus University Rotterdam, Rotterdam, The Netherlands

**Keywords:** Adolescent depressive mood, Parent-adolescent relationship, Friend-adolescent relationship, Longitudinal multilevel analyses

## Abstract

Utilizing four waves of data from 1126 secondary school Dutch adolescents (*M*age = 13.95 at the first wave; 53% boys), the current study examined the interplay between parent-adolescent and friend-adolescent relationship quality (satisfaction and conflict) in relation to adolescents’ depressive mood. Using multilevel analyses, the interacting effects of parent/friend relationship quality on depressive mood were tested at both the intra- and inter-individual level. Analyses at the intra-individual level investigated whether individual depressive mood fluctuated along with changes in their social relationships regardless of one’s general level of depressive mood; and analyses at the inter-individual level examined whether the average differences in depressive mood between adolescents were associated with different qualities of social relationships. We interpreted the patterns of interactions between parent and friend relationships using four theoretical models: the reinforcement, toxic friends, compensation, and additive model. The results demonstrate the covariation of parent- and friend- relationship quality with adolescents’ depressive mood, and highlight that parent and peer effects are not independent from each other—affirming the compensation and additive models at the intra-individual and the reinforcement and additive models at the inter-individual level. The findings highlight the robustness of the protective effects of parent and peer support and the deleterious effects of conflictual relationships for adolescent mental health. The results have implications for both the theoretical and practical design of (preventive) interventions aimed at decreasing adolescents’ depressive mood.

Social ecological theory suggests that socio-contextual systems can include risk factors for adolescent depressive mood (Earls and Carlson 2001). Relationships with parents are the most proximal context for adolescents and have critical influences—both good and bad—on the development of depression (Gutman and Eccles 2007; Steinberg 2001). At the same time, adolescence is a period when youth expand their interpersonal networks and put more emphasis on friendships (Collins 1997). During adolescence, youth form emotional bonds and deeper attachments to their friends (Collins 1997; Levpuscek 2006; Stanton-Salazar and Spina 2005). Early and middle adolescents even reported receiving more support from their friends than from their parents (Furman and Buhrmester 1992). As friends become increasingly important and have additional influences on adolescent adjustment, friendships may turn into a critical context shaping the nature of family life and moderating individual experiences in the family setting (Vandewater and Lansford 2005). The current study investigates how features of friendships moderate the effect of the parent-adolescent relationship on adolescent depressive mood.

Relationships with parents and friends can have both positive (protective) and negative (risk) associations with adolescents’ depressive mood (Cohen et al. 2015; Kenny et al. 2013; Young et al. 2005). While high satisfaction in relationships contributes to better emotional functioning including less depressive mood (Branje et al. 2010; Fanti et al. 2008; Waldrip et al. 2008), conflicts in relationships can amplify psychological problems and exacerbate depressive mood (Branje et al. 2009; Collins 1997; Demir and Urberg 2004; Laursen and Collins 1994; Rubin et al. 2004; Sentse and Laird 2010). A line of literature looking at adolescent social relationships and depression has specifically focused on the association between social support and depression. These empirical studies have robustly found a buffering effect of parent and friend support on depression, but these studies have also demonstrated that the magnitude of the main effects differed under different circumstances (Rueger et al. 2016). One way to understand the differences in the magnitude is to investigate moderators, and one of the commonly studied moderators is stress (Auerbach et al. 2011; Rueger et al. 2016). Some have argued that the buffering effect of social support is particularly important and salient in stressful circumstances, indicating that stress strengthens the (protective) association between social support and depression; however, others have found empirical support for an opposite association, showing that stress, especially high stress, undermines the protective effect of social support (Rueger et al. 2016). Thus, it is important to study the protective and risk aspects of relationships simultaneously because the risk factors of social relationships (e.g., high conflict) can be a cause of stress and may moderate the effect of the protective aspects. Furthermore, because adolescents manage and experience parent and friend relationships at the same time, stress in one relationship may also moderate the effect of the other relationship. As such, to understand the association between parent- and friend relationships and adolescent depressive mood, it is relevant to investigate both the protective (i.e., satisfaction) and risk (i.e., conflict) features of parent-adolescent relationships and how their association with adolescent depressive mood are moderated by the same features of friendships. The goal of this study is, therefore, to examine patterns in the interaction between parent and friend relationships in relation to adolescent depressive mood.

## Patterns of Interplay Between Parent and Friend Relationships

**Fig. 1:**
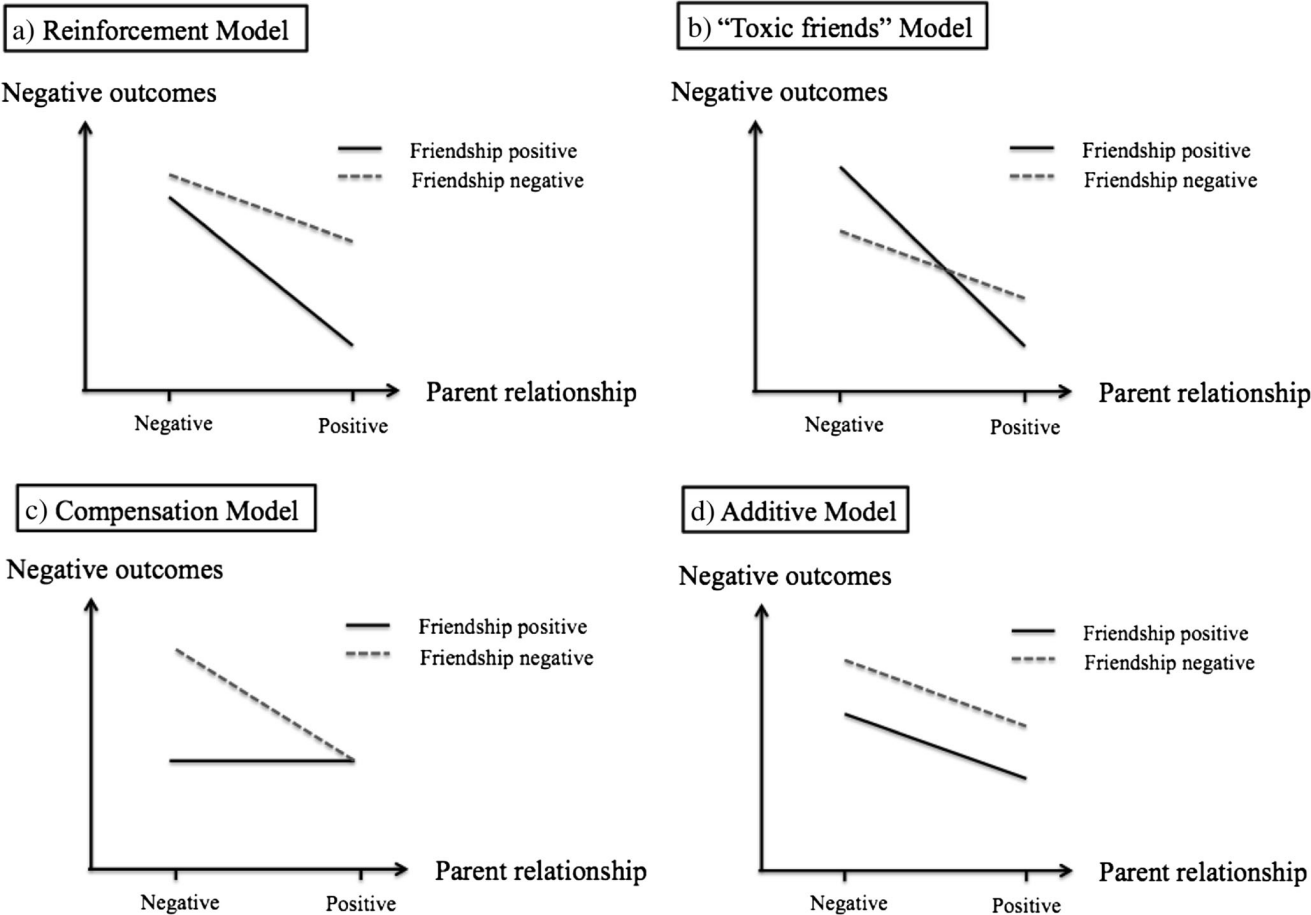
Statistical decomposition of the example interactions derived from the four theoretical models

Currently, there are four models describing patterns of how friendships may moderate the association between parent-adolescent relationships and adolescent emotional functioning (Helsen et al. 2000; Raja et al. 1992; Young et al. 2005): 1) the reinforcement model, 2) the toxic friends model, 3) the compensation model, and 4) the additive model. These models were derived from empirical research that studied the interactions between parent and friend relationship quality on various indicators of emotional functioning, such as self-esteem (e.g., Raboteg-Saric and Sakic 2014), positive self-perception (Ciairano et al. 2007), internalizing problems (Rubin et al. 2004), and depressive mood (e.g., Young et al. 2005). In the current study, we apply these models to the development of depressive mood among adolescents. Figure 1 demonstrates the statistical expressions of the four theoretical models and exemplifies how statistical results of the current study can be interpreted as support toward a certain model.

First, the *reinforcement* model refers to a mutual reinforcing effect of parent-adolescent relationship and friendship quality. It suggests that the protective effect of a good parent-adolescent relationship is stronger among the adolescents who have better friendships, and vice versa (Ciairano et al. 2007; Helsen et al. 2000; Sentse and Laird 2010). Such a synergy between the parent and friend relationship aligns with an empirically observed pattern of a reverse stress-buffering model, which found that the protective effect of family and friend support is dampened in the face of stress (Rueger et al. 2016). Since a low-satisfaction and high-conflict friendship can be stress eliciting, having such a suboptimal relationship with one’s close friend may weaken the protective association between parent-adolescent relationship and depressive mood. As such, adolescents who handle both relationships well are expected to adjust considerably better (for an example model, see Fig. 1a). Specific to the current study, having a good relationship with either parents or friends is not sufficient, rather adolescents would have to have good relationships (high satisfaction or low conflict) with both their parents and friends in order to show the lowest level of depression.

Second, a variation of the reinforcement model has been empirically observed (Raja et al. 1992; Young et al. 2005). While in the reinforcement model, it is anticipated that the most optimal emotional outcomes emerge when both parent-adolescent and friend-adolescent relationship characteristics are positive, some found that the most *negative* emotional outcomes are associated with negative parent-adolescent relationship characteristics and *positive* friend relationship characteristics (e.g., high support and attachment) (Helsen et al. 2000; Raja et al. 1992; Young et al. 2005) (for an example model, see Fig. 1b). It is worth emphasizing that a close friend (i.e., high relationship quality) does not equate to a beneficial friend (i.e., content of the friendships). Research suggests that when parent-adolescent relationships are unsatisfactory, some adolescents affiliate more with friends who are toxic^1^ (Scholte et al. 2001; Young et al. 2005). Although counterintuitive, a close friend could foster inappropriate ruminative coping styles that exacerbate depressive mood (Rose 2002; Nolen-Hoeksema 1994) or encourage (deviant) acts that elicit negative feelings (Fergusson et al. 2003; Vitaro et al. 2000). Thus, according to this model, which we refer to as the *toxic friends* model, having a negative parent-adolescent relationship may put adolescents at risk to form close but detrimental (toxic) relationships with friends with qualities that exaggerate the negative effects of poor parent-adolescent relationships (Lansford et al. 2003). Specific to the current study, adolescents would show the highest level of depressive mood when their relationship with parents is characterized as negative (low satisfaction or high conflict) and their friendships are characterized as positive (high satisfaction or low conflict).

Third, as some adolescents re-anchor their emotional and attachment needs from parents to friends (Fuligni and Eccles 1993), friendships may start to compensate for relationships with parents (Gauze et al. 1996). For example, if adolescents have a problematic relationship with their parents, but build supportive relationships with friends, these friendships may replace the socio-emotional functions of the relationship with parents (Markiewicz et al. 2006). This is referred to as the *compensation* effect (Gaertner et al. 2010; Gauze et al. 1996; Hazel et al. 2014; Helsen et al. 2000; Rubin et al. 2004; Sentse and Laird 2010; Stocker 1994) (for an example model, see Fig. 1c). Specific to the current study, a positive relationship (high satisfaction and low conflict) with either parents or friends would be sufficient for optimal emotional outcomes; and negative emotional outcomes would only be established when the relationship with both parents and friends are suboptimal (low satisfaction or high conflict).

The fourth and last model assumes that adolescents separate parents and peers as two independent social worlds (Berndt 1979; Helsen et al. 2000). Following this notion, both parents and peers affect adolescents’ emotional functioning, but influences from these two worlds are independent and can be considered as an addition on top of each other (Ciairano et al. 2007; Laible et al. 2000; Raboteg-Saric and Sakic 2014). This model is termed the *additive* model (Helsen et al. 2000) (for an example model, see Fig. 1d). It differs from the reinforcement model which suggests that the total effect of parent- and friend relationships is multiplicative and thus more than the sum of its parts. Specific to the current study, features of the parent-adolescent relationship are independent of the features of friend-adolescent relationship; and there would be no statistical interaction between the effects of these two relationships on adolescents’ depressive mood.

The four discussed models are not mutually exclusive because they could occur at the same time on different relationship aspects and at different levels of analyses. Therefore, we refer to the patterns derived from these models as alternative hypotheses. The current study examines which pattern best describes the interactions between parent and friend relationships. Because all four models could occur at the same time, it is not surprising that all four have received empirical support. Important to note, however, is that some studies only focused on protective features, such as support from parents and friends (e.g., Young et al. 2005), whereas others focused only on risk factors, such as stress and conflict in the relationships (e.g., Ciairano et al. 2007). To our knowledge, only one study examined the functions of both protective (i.e., support) and risk (i.e., conflict) features of the parent- and the friend-adolescent relationship on adolescents’ depressive mood (Sentse and Laird 2010). We interpreted their findings as support for the reinforcement and compensation model. With the current study, we extend their findings by making a distinction between the intra- and inter-individual level. Furthermore, testing the interactions between parent and friend relationships under multiple contexts enabled us to look for possible explanations to reconcile the inconsistent findings on how friendships moderate the effect of parent-adolescent relationship on adolescent depressive mood.

## Intra- and Inter-Individual Effects

As the current study utilizes four waves of data, we consider that the total variation in depressive mood is composed of two parts: 1) how on average, adolescents’ levels of depressive mood differ from one another, and this can be referred to as differences in depressive mood at the inter-individual level; 2) how each adolescent’s mood fluctuates across time regardless of his or her general level of depressive mood, and this can be referred to as fluctuation in depressive mood at the intra-individual level. The same distinction can be made on adolescents’ relationships with parents and with friends, in the sense that there is an intra- and an inter-individual component in these relationships. Within each adolescent, social ecological theory reasons that experiences in social contexts influence individual outcomes (Earls and Carlson 2001) and, hence, adolescents’ depressive mood might fluctuate as a function of the changes in their social relationships. Between adolescents, an accumulative difference in the social context might build up to differences in individual outcomes and, hence, the differences in adolescents’ depressive mood may be associated with their average level of relationship quality. In order to portray how friendships moderate the effect of parent relationships on explaining the differences between adolescents’ average level of depressive mood and on explaining the fluctuation in adolescent depressive mood, in this study, we separated the analyses into the two levels, we tested interactions between features of the parent relationships and features of the friend relationships on both the intra- and inter-individual level, and we interpreted the findings using the four models discussed above. However, we cannot form hypotheses about similarity or differences between findings at the two levels and therefore this part of the analyses is exploratory.

Conducting the analysis at both the intra- an inter-individual level has some other advantages. First, effects at the intra-individual level are not confounded by any inter-individual factors. This is because many factors, which confound inter-individual effects because they vary between individuals, are generally stable within each person over time (e.g., SES), and cannot be the third factor driving associations at the intra-individual level. Hence, while it is impossible to exhaustively control for all confounders, examining the association between relationship quality and depressive mood at the intra-individual level minimizes the effects of many inter-individual level confounders. Second, since the intra- and inter-individual levels are two different levels, an effect at the inter-individual level does not guarantee a similar effect at the intra-individual level, or even an effect at all, and vice versa (Hox 2010). However, results at both levels can be interesting because they have different implications for the design of (preventive) interventions (Vaughan et al. 2010). On the one hand, factors identified at the inter-individual level help to detect who is at risk for depression. On the other hand, findings at the intra-individual level indicate which factors have the most potential for individual changes and are thus the most effective elements worth addressing in adolescent depression prevention and interventions. In other words, between-individual factors can be used to identify *who* would most likely benefit from interventions while within-individual factors help to identify *what* should be addressed in those interventions. Multilevel analysis is the suited method for answering these research questions at the two levels.

## Method

### Sample and Participants

The present study used data from Project STARS (Studies on Trajectories of Adolescent Relationships and Sexuality), a longitudinal study among a community sample of 1297 adolescents in the Netherlands (Reitz et al. 2015). Starting from the Fall of 2011, four waves of data were collected with 6-month intervals. Participants were recruited from the last year of elementary school (6th grade) through 10th grade of secondary school. Given that the dependent variable (i.e., depressive mood) was only assessed among the 1132 secondary school students, the elementary school students were excluded from the current analyses. At wave 1, these adolescents’ ages ranged from 11 to 18 years (*M* = 13.95, *SD* = 1.18). Fifty-three percent of the sample consisted of boys. Fifty-five percent of the participating adolescents followed the high education track (i.e., senior general education or pre-university education) and 35.7% followed the low education track (i.e., prevocational education). The majority of adolescents had a Dutch ethnic background (79.2%); 11.0% had another western background; and the rest with non-western backgrounds were mainly from Surinam (2.6%), the Dutch Caribbean (1.7%), Morocco (1.3%), and Turkey (0.9%). Nineteen percent of the adolescents reported that their parents were divorced at the first wave.

The percentages of adolescents that had missing data in the constructs that we studied were 8.6%, 10.7%, 12.6%, and 19.2%, across the four waves, respectively (not accumulative). Six adolescents who had missing data in the studied constructs at all four waves were excluded from the analyses, leaving 1126 in the current analytical sample. Sixty-eight percent of adolescents completed questionnaires at all four waves. To investigate potential bias in the analyses, we compared those adolescents who provided complete response in all four waves to those who did not. Adolescents who missed participation in one or more waves were older, *t*(620.91) = 4.82, *p* < 0.001, more likely to be boys (62% vs. 48%, *χ* ^*2*^ (1, *N* = 1132) = 18.56, *p* < 0.001), and had higher levels of conflicts with parents, *t*(431.33) = 2.88, *p* = 0.004, and peers, *t*(407.64) = 2.91, *p* = 0.004; but there were no differences in mean levels of satisfaction in the parent-adolescent relationship, *t*(433.81) = −1.11, *p* = 0.269, satisfaction in the friend-adolescent relationships, *t*(1047) = −0.69, *p* = 0.489, or depressive mood, *t*(1055) = −1.30, *p* = 0.195. Representing 1126 participants who had data on the variables we studied at one or more waves, the final analyses were performed on 3966 observations. That is, to perform the longitudinal multilevel analysis, responses are translated from the individual level to the observation level. One adolescent at one wave is regarded as one observation. Thus, the responses of each adolescent can be translated to four observations at maximum.

### Procedure

Participants were recruited from four secondary schools in large cities and small municipalities in different areas of the Netherlands. Introduction of the study and the possibility of declining participation were explained to parents and adolescents through letters, brochures, and flyers. More than 93% of the approached adolescents participated in the study. The survey was conducted via online questionnaires during regularly scheduled class hours. Researchers were present to supervise the data collection. After completing the survey, adolescents received book certificates for their participation (€5, €7.5, €10, and €12.5 at wave 1, 2, 3, and 4, respectively). This study was approved by the ethics board of the Faculty of Social and Behavioural Sciences of Utrecht University.

### Measures

#### Depressive Mood

The depressive mood measure included six items from the Depressive Mood List (Kandel and Davies 1982). To curb the length of the extensive online questionnaire and to minimize potential data loss due to weariness, the scale was administered with a planned missingness design (Graham et al. 2006) at waves 1 and 2 (not waves 3 and 4): Each adolescent was randomly assigned to one of three questionnaire-groups and received three items, including one core item (“I feel unhappy and gloomy”) and two additional items (e.g., “I feel too tired to do anything”). Adolescents reported how often they experienced the indicated feelings in the previous 6 months (1 = *never*; 5 = *always*). Participants’ responses covered the full range of the scale. For the first two waves, a constructed score indicating one’s level of depressive mood was assigned to each participant by averaging the three items; average Cronbach’s alphas across the three item-combinations were 0.70 and 0.76, for waves 1 and 2, respectively. In the last two waves, all six items were used; Cronbach’s alphas were 0.84 and 0.85 at waves 3 and 4, respectively. A higher mean score indicated more depressive mood. The mean scores of depressive mood (dependent variable) were not skewed in all four waves. To estimate the percentage of the adolescents with elevated depression scores, we followed the method used by previous empirical studies (Kandel and Davies 1982; Otten et al. 2009), in which we transformed the current 5-point scale to a 3-point scale (1 = *never/almost never*, 2 = *sometimes*, 3 = *often/always*), multiplied the average score by 10 and classified adolescents as showing depressed mood if their scores were greater than 21.8. Using this cutoff value, 11.9%, 14.5%, 9.6%, and 9.7% of the adolescents were classified as depressed in the four waves, respectively. Nine adolescents were classified as depressed in all four waves.

#### Parent-Adolescent Relationship Quality

The quality of adolescents’ relationship with parents was assessed with two subscales of the Network of Relationships Inventory (NRI; Furman and Buhrmester 2009): satisfaction and conflict. Each subscale consisted of three items. A sample item for the satisfaction subscale was “How satisfied are you with the relationship with your mother (father)” and for the conflict subscale “How much do you and your mother (father) argue with each other” (1 = *little or none*; 6 = *the most*). Adolescents could choose to respond about either their mother or father, based on which parent spent the most time with them and had the most concerns for them, with 76% of adolescents reporting about their mother across all four waves, 7% reporting on their father across all four waves, and 18% changing which parent they responded about. To retain maximum data, the current study included the responses about both parents and treated them together as the parent-adolescent relationship. However, we also repeated our analyses on the mother-only subsample (76% of the total sample) as a robustness check and report the results in Footnote 4. Participants’ responses covered the full range of the scale. Mean scores over three items were taken for each subscale. A higher score on the satisfaction subscale indicated higher satisfaction with their parent relationship and a higher score on the conflict subscale indicated more conflicts with parents. Cronbach’s alphas for the four waves ranged from 0.93 to 0.95 for the satisfaction subscale, and from 0.77 to 0.81 for the conflict subscale.

#### Friendship Quality

The quality of adolescents’ friendships was also assessed with the satisfaction and conflict subscales of Network of Relationship Inventory (NRI; Furman and Buhrmester 2009). Adolescents were required to respond based on their relationship with best friends (plural). If the adolescents did not have best friends, then they should base their responses on peers who come closest to that. The scale was comparable to the measure of quality of parent-adolescent relationship, with only the objects of the items replaced by “best friends” (e.g., “How satisfied are you with the relationship with your best friends”). Participants’ responses covered the full range of the scale. A higher mean score on the satisfaction subscale indicated higher satisfaction with their friendships and a higher score on the conflict subscale indicated more conflicts with the best friends. Cronbach’s alphas for the four waves ranged from 0.91 to 0.96 for the satisfaction subscale, and from 0.74 to 0.86 for the conflict subscale.

### Analytical Plan

Using Mplus version 7.3 (Muthén and Muthén 1998–2012), we conducted a longitudinal multilevel analysis on the data which had a multilevel structure with four repeated measures nesting within individuals (Hox 2010). As discussed above, we planned to interpret out findings on both the intra- and inter-individual level using the four alternative hypotheses. Using a centering within cluster method (see Enders and Tofighi 2007 for detailed introduction of this method), we separated the main predictors, adolescent relationships with parents and friends, into two components: 1) The time-invariant component representing the average level of adolescent relationships was acquired by taking a *personal mean* across the four waves for each adolescent. These personal means were used to predict adolescents’ depressive mood at the inter-individual level. 2) The time-variant component representing the changes in adolescents’ relationships was acquired by subtracting each adolescent’s responses at each of the four waves from his or her personal mean, which resulted in four corresponding *deviance scores* indicating the fluctuation in his or her relationship qualities across time. These deviance scores were used to predict the fluctuation in adolescent depressive mood at the intra-individual. Four independent variables were handled in this way: satisfaction and conflict in the parent-adolescent relationship and in the friend-adolescent relationship.

Because the data are longitudinal, we also included a *wave* 2 variable to model the effect of time. Locating at the occasion level, the effect of the wave variable indicates an average of the linear trajectory in depressive mood of individual adolescents as a function of time. A positive significant effect would indicate that in general depressive mood increases over time. By including explanatory factors and examining whether the wave variable remains significant, we can tell if the linear change of depressive mood over time is a function of the proposed explanatory factors.

The analyses were conducted through a step-wise model building-up (in contrast to a trimming-down) technique following the standard steps of multilevel analysis (Hox 2010). That is, we began with the simplest model, and gradually developed it into a more complicated one. In total, we tested five models. Model 0 was specified as an *unconditional means model*. No predictor was included in this model. This model divides the variance of depressive mood into intra-individual and inter-individual levels. Next, we specified Model 1 as an *unconditional growth model*, in which only the wave variable was included. Controlling for the effect of time, the residual variances of depressive mood at two levels were estimated. This was used as the baseline model to which further steps were compared by assessing the decreases of residual variances. Model 2 tested the main effects of the four relationship predictors at the intra-individual level. The time-variant components of the relationship predictors—deviance scores of satisfaction and conflict in parent-adolescent relationship and in friend-adolescent relationship—were included. Model 3 extended Model 2 by including interactions between these relationship predictors. Model 3 gives the final results at the intra-individual level. The interaction terms of Model 3 tested which of the four alternative hypotheses were supported at the intra-individual level. We interpreted the results of Model 3 to answer how the interplay between parent and friend relationships predicted the fluctuation of depressive mood within adolescents.

While Models 0 to 3 were at the intra-individual level, Models 4 and 5 were at the inter-individual level. To test the main effect of the relationship predictors at the inter-individual level in Model 4, the time-invariant components of the relationship predictors—personal means of satisfaction and conflict in parent-adolescent relationship and in friend-adolescent relationship, as well as gender and age (at wave 1), were included in Model 4. Finally, Model 5 extends model 4 by including interactions between its relationship predictors. Model 5 gives the final results at the inter-individual level. The interaction terms of Model 5 tested which of the four alternative hypotheses were supported at the inter-individual level. We interpreted results of Model 5 to answer how the interplay between parent and friend relationships explained the differences in depressive mood between adolescents.

First, all interaction terms were included in the model one by one. The interactions that were significant were then simultaneously included in one model. Second, if interactions became non-significant after controlling for other interactions, they were excluded. Thus, only the interaction terms that have a significant and unique contribution were retained in the model.

To interpret the significant interactions, we decomposed them using *region of significance* analyses (Bauer and Curran 2005). Following Roisman et al.’ (2012) recommendation, we took two standard deviations above (+2*SD*) and below (−2*SD*) the mean of the independent variables and of the moderators as the ranges of analyses. To present how the effects of the independent variables are conditioned by the moderator’s level, three values of the moderator were taken for the region of significance analyses: 1) 2 *SD*s below the moderator’s mean as the lower boundary of the range, 2) 2 *SD*s above the moderator’s mean as the upper boundary of the range, and 3) the value of the moderator based upon which the effect of the independent variable is significant at *p* = 0.05 (i.e., the upper (or lower) bound of confidence interval of the effect of the independent variable hits the zero value). To add conventional simple slope analyses (Aiken and West 1991), we also calculated the effect of the independent variables conditioned by the value of the moderators at: 4) 1 *SD* above and 5) 1 *SD* below the moderator’s mean.

## Results

### Preliminary Analyses

Table 1 presents the means, standard deviations, and correlations of the explanatory factors and the dependent variable at wave 1, for boys and girls separately. Boys reported a significantly lower level of depressive mood, *t*(1008.87) = −4.68, *p* < 0.001, a lower level of friendship satisfaction, *t*(1049) = −6.92, *p* < 0.001, and a higher level of friendship conflict, *t*(1024.92) = 6.49, *p* < 0.001, than girls, but boys and girls did not differ in parent relationship satisfaction, *t*(1043) = −1.25, *p* = 0.212, and conflict, *t*(1043) = 0.75, *p* = 0.452.

**Table 1:** Means, standard deviations and correlations of key variables at wave 1, for boys and girls separately

						Boys		Girls	
Variable (range)	1	2	3	4	5	Mean	*SD*	Mean	*SD*
1. Depressive mood (1–5)	−	−0.17***	0.12**	−0.21***	0.12**	2.13^a^	0.72	2.35^a^	0.80
2. Parent satisfaction (1–6)	−0.31***	−	−0.32***	0.31***	−0.13**	4.90	0.82	4.97	0.91
3. Parent conflict (1–6)	0.32***	−0.62***	−	−0.01	0.25***	2.69	0.83	2.65	0.80
4. Friend satisfaction (1–6)	−0.19***	0.20***	−0.08	−	−0.12**	4.63^a^	0.77	4.96^a^	0.76
5. Friend conflict (1–6)	0.20***	−0.17***	0.24***	−0.25***	−	2.39^a^	0.77	2.11^a^	0.59

Correlations above diagonal refer to boys; correlations below diagonal refer to girls

** two-tailed *p* < 0.01. *** two-tailed *p* < 0.001

^a^the difference between boys and girls is significant (*p* < 0.05)

Higher satisfaction in the parent-adolescent relationship was significantly correlated with higher satisfaction with friends (Boys: *p* < 0.001; Girls: *p* < 0.001) and less conflicts with friends (Boys: *p* = 0.002; Girls: *p* < 0.001). Conflict in the parent relationship was not correlated to satisfaction with friends (Boys: *p* = 0.81; Girls: *p* = 0.07) but related to more conflicts with friends (Boys: *p* < 0.001; Girls: *p* < 0.001).

### Main Analyses

Table 2 presents the results of the multilevel analyses. Model 0 (*unconditional means model*) was the simplest model with no predictor but only a multilevel structure. The variances at both the intra-individual level, Estimate = 0.304, *SE* = 0.01, *p* < 0.001, and inter-individual level, Estimate = 0.301, *SE* = 0.02, *p* < 0.001, were significant. Thus, the IntraClass Correlation (ICC) was 50%, which indicated that the variance at the intra-individual level accounted for 50% the total variance of depressive mood. The total variance in depressive mood was evenly distributed in the intra- and inter-individual level. This suggests that there was substantial fluctuation in depressive mood within adolescents over time and also substantial differences between adolescents in their levels of depressive mood. Given that there was sufficient variation at both levels, our analyses could then examine whether our explanatory factors (parent-adolescent and friendship relationship quality) could explain both the within-adolescent fluctuation and the between-adolescent differences.

**Table 2:** Summary of model building: coefficients and standard errors for model 0 to model 5 of depressive mood

	Model 0	Model 1	Model 2	Model 3	Model 4	Model 5
Intercept	2.31 (0.02)^***^	2.20 (0.03)^***^	2.22 (0.03)^***^	2.22 (0.03)^***^	2.52 (0.29)^***^	2.32 (0.30)^***^
Intra-Individual level						
Wave		0.05 (0.01)^***^	0.04 (0.01)^***^	0.04 (0.01)^***^	0.04 (0.01)^***^	0.04 (0.01)^***^
Parent satisfaction			−0.05 (0.02)^**^	−0.05 (0.02)^**^	−0.05 (0.02)^**^	−0.05 (0.02)^**^
Parent Conflict			0.08 (0.02)^***^	0.09 (0.02)^***^	0.09 (0.02)^***^	0.09 (0.02)^***^
Friend Satisfaction			−0.02 (0.02)	−0.03 (0.02)	−0.03 (0.02)	−0.03 (0.02)
Friend Conflict			0.06 (0.02)^***^	0.06 (0.02)^***^	0.06 (0.02)^***^	0.06 (0.02)^***^
Parent Satisfaction × Friend Conflict				−0.07 (0.02)^**^	−0.07 (0.02)^**^	−0.07 (0.02)^**^
Parent Conflict × Friend Satisfaction				−0.08 (0.03)^**^	−0.08 (0.03)^**^	−0.08 (0.03)^**^
Inter-Individual Level						
Gender					0.39 (0.03)^***^	0.39 (0.03)^***^
Age at wave 1					−0.01 (0.01)	−0.00 (0.01)
Parent satisfaction					−0.11 (0.03)^***^	−0.10 (0.03)^***^
Parent Conflict					0.22 (0.03)^***^	0.24 (0.03)^***^
Friend Satisfaction					−0.16 (0.03)^***^	−0.15 (0.03)^***^
Friend Conflict					0.12 (0.03)^***^	0.14 (0.03)^***^
Parent Conflict × Friend Conflict						−0.11 (0.04)^**^
Residual Variance						
Intra-individual level	0.304 (0.01)	0.301 (0.01)	0.294 (0.01)	0.292 (0.01)	0.292 (0.01)	0.292 (0.01)
Inter-individual level	0.301 (0.02)	0.301 (0.02)	0.303 (0.02)	0.304 (0.02)	0.201 (0.01)	0.199 (0.01)

Gender (0 = boys; 1 = girls)

** two-tailed *p* < 0.01. *** two-tailed *p* < 0.001

Model 1 (*unconditional growth model*) included only one predictor—the *wave* variable. This model showed the amount of change in depressive mood as a function of time. Residual variance at the intra-individual level decreased from 0.304 (Model 0) to 0.301, which indicated that only a small proportion (1%) of the within-adolescent fluctuation of depressive mood could be modeled as a linear increase over time. The residual variances (at both levels) of Model 1 served as the baseline upon which the improvement of all further models was evaluated.

Model 2 included the deviance scores of the four explanatory variables at the intra-individual level. Results of this model indicated whether adolescents’ levels of depressive mood fluctuated along the changes in their relationships with parents and friends. The results showed that higher levels of conflict with a parent, Estimate = 0.08, *SE* = 0.02, *p* < 0.001, and with a friend, Estimate = 0.06, *SE* = 0.02, *p* < 0.001, were associated with a higher level of depressive mood, whereas a higher level satisfaction in the relationship with a parent was related to a lower level of depressive mood, Estimate = −0.05, *SE* = 0.02, *p* = 0.002. However, satisfaction in friendships was not significantly related to depressive mood, Estimate = −0.02, *SE* = 0.02, *p* = 0.223. See Table 2.

Model 3 included interaction terms between the four relationship factors at the intra-individual level. These interactions were first incorporated one by one. The interaction between parent conflict and friend conflict was not significant, Estimate = −0.01, *SE* = 0.02, *p* = 0.679, but the other three interactions did show significant effects. Parent satisfaction interacted with friend satisfaction, Estimate = −0.06, *SE* = 0.02, *p* = 0.013; parent satisfaction interacted with friend conflict, Estimate = −0.08, *SE* = 0.02, *p* = 0.004; and finally, parent conflict interacted with friend satisfaction, Estimate = −0.10, *SE* = 0.03, *p* < 0.001. The three significant interactions were incorporated into the same model, two of which remained significant and were retained in the model: The interaction between satisfaction in the parent relationship and conflict in the friend relationship, and between conflict in the parent relationship and satisfaction in the friend relationship (estimates presented in Table 2). This model was accepted as the final Model 3, upon which further steps were built.

**Fig. 2:**
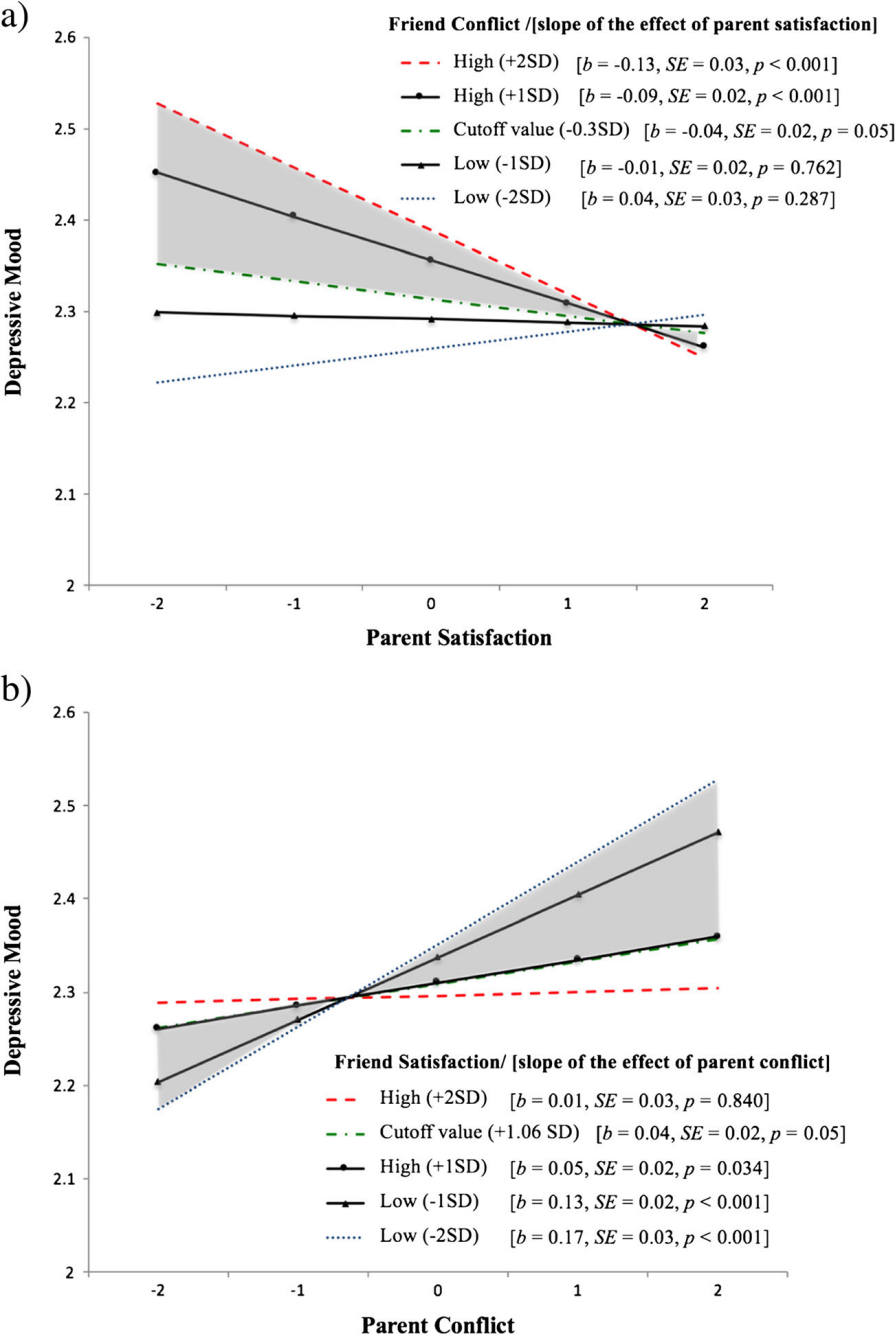
Intra-individual differences in adolescent depressive mood as a function of the interaction between parent satisfaction and friend conflict (panel **a**) and between parent conflict and friend satisfaction (panel **b**). The *red-dashed*, *black (triangle)*, *black (circle)*, and *blue-dotted line* represent the effect of the independent variable (IV) when the moderator is two standard deviations (SD) above, one SD above, one SD below, and two SD below its mean, respectively. The *green-dashed-dotted line* represents the effect of IV when the *p*-value corresponding to its significance is 0.05. The *shaded area* indicates the region of significance

The significant interaction between parent satisfaction and friend conflict is presented in Fig. 2.^3^ The red-dashed line and the blue-dotted line depict the slope of parent satisfaction, when friend conflict was 2*SD*s above and below its mean, respectively. The green-dashed-dotted line depicts the effect of parent satisfaction at *p* = 0.05, which empirically occurred when the value of friend conflict was 0.3*SD* below its mean. The region of significance is indicated by the shaded area between the red and green lines. Additionally, the two black-solid lines demonstrate the results of the simple slope analysis; that is, the effects of parent satisfaction on depressive mood when friend conflict was 1*SD* above and below its mean. The results show that when friend conflict was high, parent satisfaction was negatively associated with depressive mood. Conversely, when friend conflict was low, parent satisfaction was not significantly associated with depressive mood.

The significant interaction between parent conflict and friend satisfaction is depicted in Fig. 2. Likewise, we drew five lines to present the interaction effect. This result shows that when friend satisfaction was high, parent conflict was not significantly associated with depressive mood. Conversely, when friend satisfaction was low, parent conflict was positively associated with depressive mood.

Starting from Model 4, the analyses aimed at explaining variance in depressive mood between adolescents. Model 4 included predictors at the inter-individual level—gender, age, and personal means of the explanatory factors—to explain the difference of depressive mood *between* adolescents. The results demonstrated that girls were more depressed than boys (*p* < 0.001), those with higher satisfaction in the parent relationship (*p* < 0.001) and in the friend relationship (*p* < 0.001) reported a lower level of depressive mood, and those with higher conflict in the parent relationship (*p* < 0.001) and in the friend relationship (*p* < 0.001) reported higher levels of depressive mood.

Model 5,^4^ again, included interaction terms between the four explanatory factors at the inter-individual level one by one. Only one out of the four interaction terms was found to be statistically significant, which was the one between parent and friend conflict (see Table 2; *p* = 0.002). The interactions between parent and friend satisfaction, Estimate = −0.02, *SE* = 0.03, *p* = 0.598, between parent satisfaction and friend conflict, Estimate = 0.06, *SE* = 0.03, *p* = 0.065, and between parent conflict and friend satisfaction, Estimate = −0.03, *SE* = 0.04, *p* = 0.481, were not significant.

**Fig. 3:**
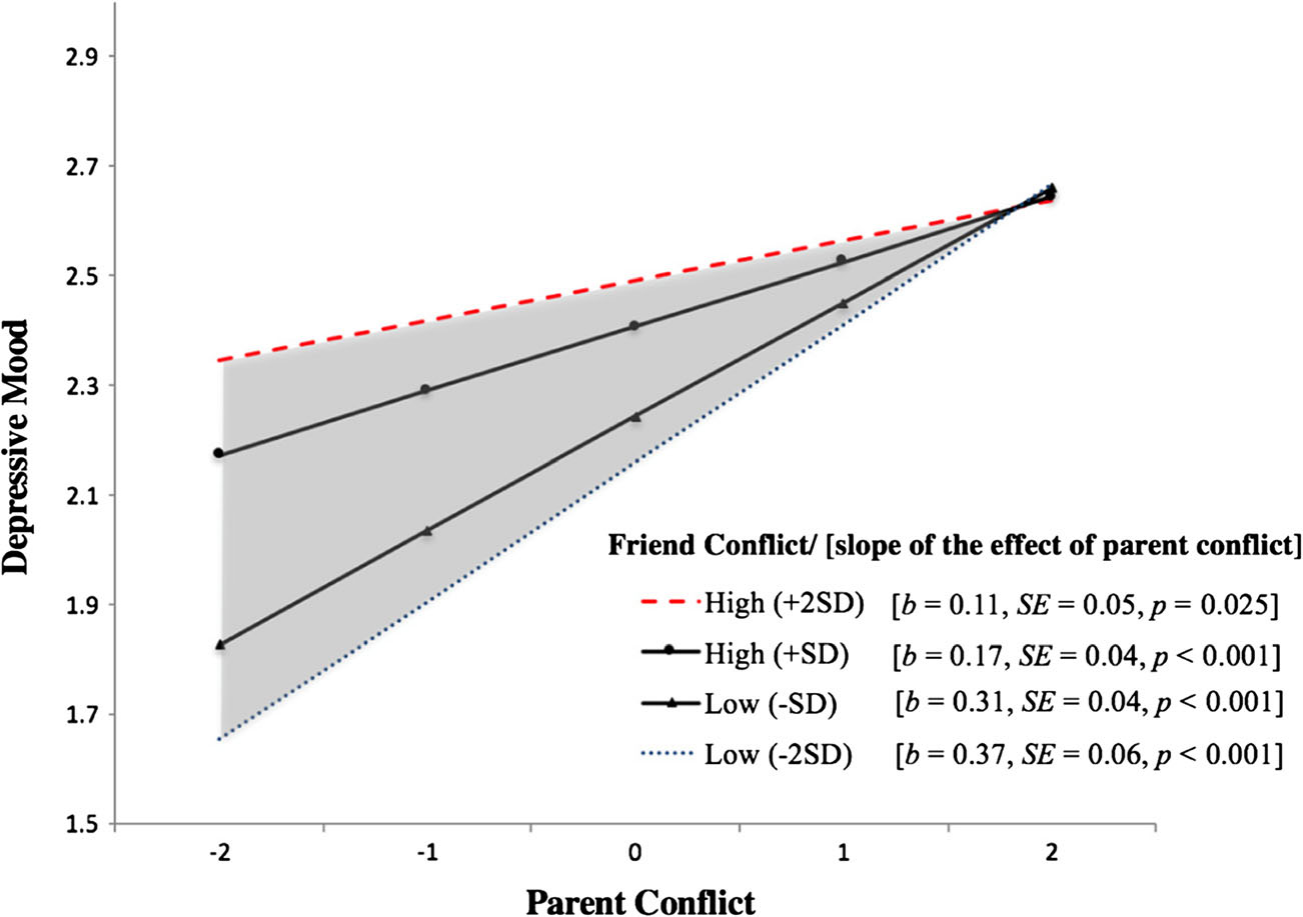
Inter-individual differences in adolescent depressive mood as a function of the interaction between parent conflict and friend conflict. The *red-dashed*, *black (triangle)*, *black (circle)*, and *blue-dotted line* represent the effect of the independent variable (IV) when the moderator is two standard deviations (SD) above, one SD above, one SD below, and two SD below its mean, respectively. The *shaded area* indicates the region of significance

The significant interaction between parent and friend conflict at the inter-individual level is presented in Fig. 3. A line indicating the cutoff value (the green-dashed-dotted line) does not exist in this figure because, as shown by the shaded area, the effect of parent conflict on depressive mood remained positive and significant for all values of friend conflict within the range of ±2*SD*s. Despite the consistent significance, this interaction suggests that the association between parent conflict and depressive mood was stronger when friend conflict was low than when it was high, as indicated by the blue-dotted line being steeper than the red-dashed line.

As can be seen in Table 2, when comparing the final model (Model 5) with the baseline model (Model 1), the residual variance at the intra-individual level decreased from 0.301 to 0.292. Thus, our final model explained 3% of the variance of the within-adolescent fluctuation in depressive mood in addition to its linear increase over time. The effect of the *wave* variable decreased slightly but remained significant in the final model, indicating that the parent and friend relationship factors did not completely explain the linear increase in depressive mood over time. At the inter-individual level, residual variance decreased from 0.301 to 0.199. That is, our final model explained 34% of the variance of the between-adolescent difference in depressive mood, indicating that the parent and friend relationship factors accounted for one third of the differences in depressive mood between adolescents.

## Discussion

The current study investigated the interplay between conflict and satisfaction in parent and friend relationships in relation to adolescents’ depressive mood. Based on which interaction is significant and how it relates to Fig. 1 (the statistical expression of the four theories), we interpret the final results derived in Model 3 for the intra-individual level and in Model 5 for the inter-individual level using the four theoretical models: the reinforcement, toxic friends, compensation, and additive model (Helsen et al. 2000; Raja et al. 1992; Young et al. 2005). We found support for different models at the within- and between- person levels.

In the prediction of the fluctuation in depressive mood within adolescents (intra-individual level) our findings support both the compensation and additive models. Having low-conflict and highly satisfactory friendships alleviated the adverse effect of a dissatisfactory and high-conflict parent relationships on depressive mood. Such findings confirm the compensation model (also see Gaertner et al. 2010; Hazel et al. 2014; Rubin et al. 2004) because positive characteristics of friendships compensated for the adverse effects of suboptimal parent-adolescent relationships, and the most depressive mood emerged only when both relationships showed negative characteristics. These results suggest that functions of parents and friend relationships can replace one another to regulate depressive mood within adolescents (Gauze et al. 1996; Levpuscek 2006; Sentse et al. 2010) and also confirm Berndt’s (1979) suggestions that (at a certain phase of adolescence) parents and friends belong to separate worlds, and thus some features of these two relationships independently relate to adolescent functioning.

To explain the differences in depressive mood between adolescents (inter-individual level), both the additive model and reinforcement models were supported. The protective effect of satisfaction with the parent relationship on depressive mood was independent from friendship satisfaction or conflict. Similarly, the effect of parent conflict was independent from friendship satisfaction. However, we also found that the protective effect of low conflict in parent relationships was strengthened by low conflict in friendships. In other words, low conflict in either parent or friend relationship by itself could not guarantee the lowest level of depressive mood, conflict needs to be minimized in *both* relational contexts for adolescents to demonstrate the best outcome.

Concluding, our findings underline that, to lower depressive mood *within* adolescents, improving at least one characteristic of friendships (i.e., increasing satisfaction or reducing conflict) may compensate for adverse effects of the other characteristic in the parent relationship (high conflict and low satisfaction). In addition, to understand why some adolescents are more troubled by depressive mood than others, our findings at the inter-individual level show that low conflict in both the parent and friend relationship are necessary conditions for an optimally low level of depressive mood.

Our results demonstrate the conceptual independence between the intra- and inter-individual level (Hox 2010; Vaughan et al. 2010). The fact that there was substantial variance of depressive mood at both levels suggests that two sets of explanatory factors are needed: One set at the inter-individual level that explains *who*, on average, is more depressed than others; the other set at the intra-individual level that explains *why*, within adolescents, depressive mood fluctuates over time. Furthermore, the finding that the two levels show different results is in agreement with Vaughan et al. (2010) who found that adolescents’ age influenced the effect of maternal support on depressive symptoms differently for boys and girls (i.e., a three-way interaction) at the between- but not at the within-adolescent level.

The current study offers a new perspective that may help to reconcile the inconsistent empirical findings on the interplay between parent and friend relationship characteristics in relation to adolescents’ emotional functioning: It is possible that the interplay differs at the intra- and inter-individual level. For individual adolescents, one may compensate for the deleterious impact of a poor parent relationship by seeking comfort from friends. However, when depressive mood is compared between adolescents, those who have favorable relationships with both parents and friends are likely to be better adjusted than those who have a favorable relationship with just one or the other. In other words, the compensation effects mostly occur at the intra-individual level, whereas the reinforcement effects mostly occur at the inter-individual level. Such inferences are compatible with the theoretical reasoning of the compensation model which is based on the adolescent individuation process (Fuligni and Eccles 1993; Gauze et al. 1996; Levpuscek 2006; Markiewicz et al. 2006) in which emotional needs are re-anchored from parents to friends.

The reinforcement model, instead, refers to a synergy between the effects of parent and friend relationships. Such a synergic interplay is in agreement with an *amplification* pattern observed on influences of different community ties on children. Children who are already advantaged in social capital by their families (have authoritative parents themselves) benefit the most from positive friendships (Fletcher et al. 1995). In other words, good friendships reinforce the existing favorable features of parent relationships and make those who are psychologically strong even better (Steinberg 2001). Further studies are needed to validate whether this kind of pattern (i.e., reinforcement/ amplification/ synergy) on the effects of parent and peers indeed locates at the between adolescent level.

It is noteworthy that our study did not support the toxic friends model, which is an empirically observed pattern that links the most problematic emotional status to negative parent-adolescent relationship characteristics and *positive* friendship characteristics (Helsen et al. 2000; Raja et al. 1992; Young et al. 2005). One possibility for the current absence of empirical support is that adolescents in our sample were not affiliating with highly toxic friends. Another potential reason is that, while we examined satisfaction and conflict in the parent and friend relationships, the previous research which found the toxic friends pattern studied social support (Helsen et al. 2000; Young et al. 2005) and attachment (Raja et al. 1992). To adequately test the negative (toxic) influences of friends, future studies need to not only ask about friendship quality, but also other friendship characteristics that may indicate the toxicity of the friendships, such as friends’ coping style, mental health, and deviant behaviors. Comparing the effects of close relationships with wholesome friends to those with toxic friends will shed light on how friendship’s influences may differ by the nature of the friends.

### Limitations

The current study has a few limitations worth addressing. First, the current analyses, although conducted across multiple waves, were cross-sectional in nature and therefore the direction of effects cannot be determined. It could be that poor relationships with parents and friends lead to a higher level of depressive mood; but it is also possible that more depressed adolescents create and/or perceive more negative relationships with parents and friends (Reitz et al. 2006); or the relationships are bidirectional (Bell 1968). Future studies that implement interventions for depressed adolescents may provide valuable insights about the directionality between the observed relationships.

Second, although the current use of adolescent self-report measures has the advantage of knowing how adolescents subjectively perceive their relationships, this prevents us from objectively examining adolescents’ relationships with parents and friends. Future studies may use multi-informant designs or observational designs to capture adolescents’ relationship with their parents and friends and compare the obtained results to those found with the adolescents’ self-report. Such comparisons help to tease apart the potential biases in how adolescents’ mood affects their perceptions of relationship quality.

Third, due to the planned missingness design (Graham et al. 2006), three versions of the depressive mood scale were used to collect data on adolescents’ depressive mood at waves 1 and 2—adolescents were randomly assigned to one of the three versions. However, although adolescents responded to slightly different items at these two waves, the correlations between the three versions of measurement were high (*r* ranges from 0.74 to 0.91). In addition, at all four waves, less than 15% of the adolescents passed the suggested cut-off point to be classified as having elevated depressed mood. As our sample is a non-clinical sample, the current findings might be less generalizable to severely depressed adolescents.

Fourth, the current study analyzed adolescents’ responses about their relationship with either their mother or father. The main findings at the intra-individual level were not robust among the subsample of adolescents who consistently reported mother-adolescent relationships across four waves. Although this may be due to the 24% reduction in sample size, we cannot draw definitive conclusions on the basis of our findings. Relationships with fathers and mothers may have different functions (Duchesne and Ratelle 2014; Rubin et al. 2004) and combining the father- and mother-adolescent relationships may conceal some nuances with regard to the differences in their interplay with friendships. Investigating the different roles of relationships with mothers and fathers in the development of depression during adolescence is an important direction for future research. Studies that collect data about adolescents’ relationship with both parents could make meaningful comparisons between effects of these two relationships or between same-sex and other-sex parent-child dyads. Additionally, adolescents in the current sample were in general quite satisfied with their parent relationships, so our findings might be less generalizable to adolescents whose parent relationships are highly dissatisfactory.

Fifth, friendships were operationalized as relationships with best friends in the current study and therefore the role of general peer relationships was not investigated. Nonetheless, our finding that friendships can compensate for poor parent-adolescent relationships is noteworthy. These results underscore the special role that best friends play in adolescents’ mental health and as is consistent with other research on adolescents (e.g., Wilkinson 2010). Finally, the current sample is relatively homogenous as the majority of our sample had a Western background (90%) as compared to the general Dutch population (84%) (Statistics Netherlands 2013).

### Practical Relevance

In spite of these limitations, the current findings have meaningful implications for (preventive) interventions and clinical practice. Our findings echo one recommendation of Horowitz and Garber (2006) that more theories recognizing the role of multiple interacting intrapersonal and interpersonal factors are needed to guide the design of prevention programs. Our results at the inter-individual level help to identify *who* is most at risk of depressive mood (Vaughan et al. 2010). Most noticeably, adolescents who have high conflict with either parents or friends are likely to have high levels of depressive mood, while in terms of satisfaction in relationships, adolescents with low satisfaction in both their parent and friendship would be most at risk for depressive mood.

The intra-individual results allow us to make inferences about *how* to effectively intervene in the development of adolescents’ depressive mood. The current findings underscore previous recommendations that parents and adolescents need to work together to improve the quality of their relationships (e.g., Connell and Dishion 2008; Perrino et al. 2015). Moreover, our results also suggest that helping adolescents to build conflict-free friendships or to effectively cope with conflicts in friendships may buffer and even compensate for the negative effects of low satisfaction in the parent-adolescent relationship. This is particularly relevant in instances where parents are unwilling to be part of the therapeutic process. Moreover, improving the satisfaction of friendships may help to combat the negative impact of conflicts with parents. Our suggestions align with the efficacy of intervention programs which teach adolescents general interpersonal skills. Adolescents benefit from learning conflict management, perspective taking, emotion regulation, effective communication, and skills to broaden social support (e.g., Shochet et al. 2001; Young et al. 2006). In addition to improving adolescent depressive mood by targeting the parent relationship (Horowitz and Garber 2006; Lewinsohn and Clarke 1999), our findings highlight the importance of giving adolescents tools to develop alternative sources of social and emotional support (such as friendships) in order to prevent the development of depressive mood and stimulate healthy and positive emotional well-being.

## Footnotes

1 The term used by Scholte et al. (2001) and Young et al. (2005) is deviant friend. We chose to use the term toxic friend because the friends are not necessarily deviant friends, but may also influences adolescents though their depressive mood or ruminative coping styles.

2 Our dataset has a structure that each individual corresponds to four cases, while each case indicates the individual at one wave. The wave variable indicates the wave to which each case corresponds, and the variable has at most four values (i.e., 1, 2, 3, and 4; some adolescents have missing data at one or more waves).

3 The exact values are taken from the final model (Model 5). There are some differences in the values between Model 3 and 5, but the differences cannot be seen at the two-decimal level.

4 We repeated our analyses among a subsample of adolescents who consistently reported about their mothers in all four waves (76% of the full sample). In terms of direction and significance, the main effects of parent and friend relationships at both intra- and inter-individual levels were the same as the results for the full sample. The interaction at the inter-individual level also remained significant with the same direction (parent conflict × friend conflict, Estimate = −0.141, *SE* = 0.048, *p* = 0.003). However, the two interactions that were found at the intra-individual level became non-significant (parent satisfaction × friend conflict: Estimate = −0.025, *SE* = 0.032, *p* = 0.448; and parent conflict × friend satisfaction: Estimate = −0.053, *SE* = 0.035, *p* = 0.129).
